# Shark Cartilage-Derived Anti-Angiogenic Peptide Inhibits Corneal Neovascularization

**DOI:** 10.3390/bioengineering11070693

**Published:** 2024-07-09

**Authors:** Yunxian Li, Aoke Chen, An Hong, Sheng Xiong, Xiaojia Chen, Qiuling Xie

**Affiliations:** 1 College of Life Science and Technology, Jinan University, Guangzhou 510632, China; liyunxian327@163.com (Y.L.); tha@jnu.edu.cn (A.H.); xsh_jnu@hotmail.com (S.X.); 2 National Engineering Research Center of Genetic Medicine, Guangzhou 510632, China; 13710815644@163.com; 3 Guangdong Jida Engineering Research Center of Genetic Medicine Co., Ltd., Guangzhou 510535, China

**Keywords:** shark cartilage, polypeptide, corneal neovascularization, anti-angiogenesis

## Abstract

Corneal neovascularization is a significant cause of vision loss, often resulting in corneal clouding and chronic inflammation. Shark cartilage is widely recognized as a significant natural source of anti-angiogenic compounds. Our previous studies have shown that a polypeptide from white-spotted catshark (*Chiloscyllium plagiosum* Bonnet) has the potential to inhibit the angiogenesis of breast tumors. This study applied this peptide (SAIF) to a corneal alkali injury model to assess its effect on corneal neovascularization. Results revealed that SAIF inhibits endothelial cell proliferation, migration, and tube formation. SAIF inhibited VEGF-induced angiogenesis in the matrigel plug. Using the corneal alkali injury model, SAIF significantly inhibited corneal vascular neovascularization in mice. We found that SAIF not only significantly inhibited the upregulation of pro-angiogenic factors such as VEGF, bFGF, and PDGF expression induced by alkali injury, but also promoted the expression of anti-angiogenesis factor PEDF. Moreover, we also analyzed the MMPs and TIMPs involved in extracellular matrix (ECM) remodeling, angiogenesis, and lymphangiogenesis. We found that SAIF treatment inhibited the expression of pro-angiogenic factors like MMP1, MMP2, MMP3, MMP9, MMP13, and MMP14, and promoted the expression of anti-angiogenesis factors such as MMP7, TIMP1, TIMP2, and TIMP3. In conclusion, SAIF acts as an anti-angiogenic factor to inhibit the proliferation, migration, and tube formation of endothelial cells, inhibit pro-angiogenic factors, promote anti-angiogenic factors, and regulate the expression of MMPs, ultimately inhibiting corneal neovascularization.

## 1. Introduction

The cornea is avascular tissue that is not permeated by capillaries or other blood vessels, which is one of the main reasons for the corneal transparency [1,2]. Physiologically, the corneal avascular state is maintained by a delicate balance between low levels of pro-angiogenic factors and high levels of anti-angiogenic factors. However, this balance can be disrupted under certain pathological conditions like corneal chemical injury or infection, resulting in the invasion of capillaries, which leads to vision loss, corneal neovascularization, and blindness [3,4]. Most clinical treatments for corneal neovascularization are surgical treatments such as vessel occlusion by topical ascorbic acid, cautery, or photodynamic therapy as well as cryotherapy, which may cause multiple complications, although it is associated with moderate improvement. The most effective treatment currently available is subconjunctival injection of VEGF inhibitors to inhibit corneal angiogenesis directly [5]. There are several marketed anti-VEGF antibodies for treating corneal neovascularization, including bevacizumab, ranibizumab, and abciximab [6,7]. These drugs block all VEGF activities by inhibiting VEGF/VEGFR interactions [8]. However, the high molecular weight of antibodies makes them difficult to absorb, and a certain degree of drug resistance may develop with prolonged use [9].

Shark cartilage has emerged as a significant area of research for inhibiting neovascularization among the discovered substances. Cartilage, a highly specialized connective tissue, distinguishes itself by the spontaneous disappearance of blood vessels formed during embryonic development after birth [10,11]. An angiogenesis inhibitory factor was isolated from cartilage, which can strongly inhibit tumor-induced capillary proliferation [12]. Compared with small amounts in mammals [13], sharks have a high proportion of cartilage, so they are expected to be an ideal source of cartilage for inhibiting angiogenesis and tumors [14]. Sheu et al. identified a shark cartilage-derived angiogenesis inhibitor, called U-995, which significantly inhibited cell migration and HUVEC proliferation [15]. Shark cartilage extracts have been shown to disrupt tumor necrosis factor-α(TNFα) and basic fibroblast growth factor (bFGF)-induced angiogenesis [15,16] and to prevent the growth of various tumor cells [17,18]. Shark cartilage extract also acts as a mammalian collagenase inhibitor to inhibit the activity of proteases that lyse the basement membrane of vascular endothelial cells [19].

In the previous study, we cloned a 183-amino acid protein of shark vascular inhibitory factor from a white-spotted bamboo shark (*Chiloscyllium plagiosum*, Bonnet), and a peptide of 33-amino acid based on the active domain of this protein was cloned [17,20]. Our previous study showed that this peptide could inhibit the angiogenesis of breast tumors [17]. While most studies on shark cartilage-derived angiogenesis inhibitors have emphasized tumor angiogenesis, a gap exists in our understanding of their impact on corneal vascular neovascularization. In this research, using the corneal alkali burn model, we investigate the inhibitory effect of this 33-amino acid peptide (SAIF) on corneal neovascularization.

## 2. Materials and Methods

### 2.1. Cell Proliferation Assay

Human umbilical vein endothelial cells (HUVEC) were cultured at 37 °C in a humidified atmosphere with 5% CO_2_ in Dulbecco’s Modified Eagle’s Medium (DMEM) (Gibco, Grand Island, NE, USA) containing 10% fetal bovine serum (FBS) (NATOCOR). Upon reaching 80–90% confluence, the HUVEC was digested using 0.25% trypsin-ethylenediaminetetraacetic acid (EDTA) (Gibco, Grand Island, NE, USA), followed by centrifugation and resuspension in DMEM.

HUVEC cells were seeded in 96-well plates at a concentration of 3000 cells per well and maintained in DMEM containing 10% FBS for 12 h. Subsequently, the cells were starved with serum-free medium for 2 h. SAIF was diluted with DMEM medium containing 0.1% FBS to different concentrations of 0.05, 0.1, 0.15, and 0.2 mg/mL. The cells were then exposed to different concentrations of SAIF for 48 h, with DMEM containing 0.1% FBS as the control. Following the treatment, the 96-well plate was incubated at 37 °C for 2 h after 10 μL of CCK-8 solution was added to each well, and then the OD_450_ and OD_630_ for each well were read using a microplate reader, OD = OD_450_- OD_630_. Cell viability (%) = OD (SAIF group)/OD (control group) × 100%.

### 2.2. Cell Migration Assay

HUVEC cells were seeded into 6-well plates at a density of 1 × 10^6^ cells/well and incubated in DMEM supplemented with 10% FBS for 12 h. The cells were starved for two h in a serum-free media once the cell confluence reached 90%. The monolayer of cells was scraped using a sterile 200 μL pipette tip and rinsed three times with 1× PBS to eliminate non-adherent cells. Cells were then stimulated for 48 h with 0.2 mg/mL SAIF, and DMEM with 0.1% FBS serving as the control. Photos were taken at 0, 16, and 24 h after the treatment. Cell migration rate (%) = [0 h scratch width − (16, 24) h scratch width]/0 h scratch width × 100%.

### 2.3. Transwell Assay

Firstly, 24-well plates filled with 600 μL of DMEM medium containing 20% FBS, and a 0.8 μm Transwell chamber was inserted into each well (Corning, New York, NY, USA). SAIF diluted to 0.2 mg/mL with DMEM medium. Afterward, cells were resuspended in DMEM medium with 0.2 mg/mL SAIF and added to the chambers at a concentration of 5 × 10^4^ cells/well, and DMEM medium was used as the control. The 24-well plates were incubated at 37 °C in a humidified atmosphere containing 5% CO_2_ for 24 h. Following a 15 min fixation in formaldehyde, the cells were stained with crystal violet. The number of migrated cells was observed and counted under a microscope.

### 2.4. Matrigel Tube Formation Assay 

The matrix gel (BD, Franklin Lakes, NJ, USA) was thawed at 4 °C in advance and added 50 μL of thawed matrix gel to the pre-cooled 96-well plate. Subsequently, the 96-well plate was equilibrated at 4 °C for 10 min to ensure even spread of the matrix gel, after which it was placed in the incubator at 37 °C for 30 min to solidify the matrix gel. HUVEC cells were pre-treated for 24 h with 0.2 mg/mL SAIF-containing and drug-free DMEM + 1% FBS medium, followed by resuspension of the cells using DMEM medium containing 0.2 mg/mL SAIF, and DMEM medium used as the control. Cells (50 μL/well) were added to 96-well plates with matrix gel at a density of 2.5 × 10^4^ cells/well, then incubated in a 37 °C, 5% CO_2_ incubator for 6 h. Five fields of view/well were randomly selected and photographed under a microscope. The number of tubes counted by Image J 1.52a software.

### 2.5. Matrigel Plug Assay

All animal study protocols are approved by the Laboratory Animal Ethics Committee of Jinan University (Ethics Code: 20220612-01). The male Babl/c mice (6–7 weeks old, 20–25 g weight) were purchased from Yoda Biotechnology Company (Guangzhou, China). The matrix gel (BD, USA) was thawed at 4 °C in advance. The mice were randomly divided into three groups (*n* = 5 mice each): the blank control group (0.9% NaCl); the positive control group (250 ng/mL VEGF + 50 IU/mL heparin); the SAIF group (0.2 mg/mL SAIF + 250 ng/mL VEGF + 50 IU/mL heparin). Mice anesthetized with an intraperitoneal dose of 1% sodium pentobarbital (40–50 mg/kg), then the matrix gel was slowly injected subcutaneously into the dorsal side of the hind limbs.

Seven days after the matrix gel injection, the mice were euthanized, and the matrix gel was pictured and then fixed in 4% paraformaldehyde for subsequent experiments.

### 2.6. Corneal Alkali Injury Model

The male Babl/c mice (6–7 weeks old, 20–25 g weight) were anesthetized with an intraperitoneal dose of 1% sodium pentobarbital (40–50 mg/kg) and ocular surface anesthesia through Oxybuprocaine hydrochloride eye drops (1 drop/dose). Subsequently, a circular filter paper with a diameter of 2 mm was soaked in 2 μL of 1 mol/L sodium hydroxide for 10 s. After the mouse cornea was exposed to this filter paper for 30 s, the alkali-burned ocular surface was promptly and thoroughly washed with saline (20 mL). 

Mice with an eye injury were randomly divided into four groups (*n* = 9): the negative control group (0.9% NaCl, 20 μL); the positive control group (Ranibizumab, 10 mg/mL, 10 μL); the high-dose group: 60 μg of SAIF (3 mg/mL, 20 μL); the low-dose group: 12 μg of SAIF (0.6 mg/mL, 20 μL). The blank control group received no treatment. After corneal alkali injury, the drug was administered as a single subconjunctival injection using a G32 100U insulin syringe. Ofloxacin eye drops were applied to the mouse cornea for infection prevention.

Mouse corneas were imaged at 4 and 7 d post-treatment, and the area of corneal neovascular was measured using Image J 1.52a software. The mice were euthanized 7 days after drug treatment, and their corneas were taken for further analysis.

### 2.7. Immunohistochemistry

Mouse corneal sections were fixed in 4% paraformaldehyde for 10 min. The slides were then closed with 10 mM sodium phosphate buffer containing 2% BSA for 1 h at room temperature. Then, the sections were incubated with primary antibodies for 1 h at room temperature. The primary antibodies used were anti-CD31 (1:5000, Proteintech, Rosemont, IL, USA), anti-bFGF (1:200, Proteintech, USA), anti-VEGF (1:200, Proteintech, USA), anti-PDGF (1:100, Proteintech, USA), and anti-PEDF (1:200, Abcam, Cambridge, UK). The sections were then incubated with anti-rabbit IgG (1:3000, Proteintech, USA) containing horseradish peroxidase. The sections were visualized with 3,3′-diaminobenzidine tetrahydrochloride (Shenggong, Shanghai, China). The sections were then counterstained with hematoxylin. Quantitative analysis of immunostaining was performed using Image J 1.52a software. One corneal section was randomly selected from each mouse, and three samples with the same high-magnification positive field of view were taken from each section for evaluation. The integrated optical density (IOD) of all positive staining in each field of view and area of interest (AOI) was measured. IOD was used to assess the area and intensity of positive staining. The mean optical density (MOD) was used to represent the concentration of specific proteins per unit area. MD = IOD/AOI.

### 2.8. Real-Time Fluorescence Quantitative PCR

Total RNA was isolated from corneal tissue using the RNAeasy Animal RNA Extraction Kit (Beyotime, Shanghai, China). Next, complementary DNA was synthesized according to the manufacturer’s instructions using the PrimeScript 1st Strand cDNA Synthesis Kit (Takara Bio, Otsu, Japan). The ChamQ Universal SYBR^®^ qPCR Master Mix (Vazyme, Nanjing, China) was used for the Real-time quantitative PCR. The comparative CT approach was used to perform the relative quantification, with GAPDH as the internal reference gene.

### 2.9. Statistical Analysis

Statistical analyses were performed by GraphPad Prism 7 software. Data were expressed as mean ± standard deviation (SD). Values for differences between groups were measured using Student’s t for comparison or one-way ANOVA for multiple comparisons. A value of *p* < 0.05 was considered statistically significant.

## 3. Results

### 3.1. SAIF Inhibits the Proliferation and Migration of HUVEC Cells

**Figure 1:**
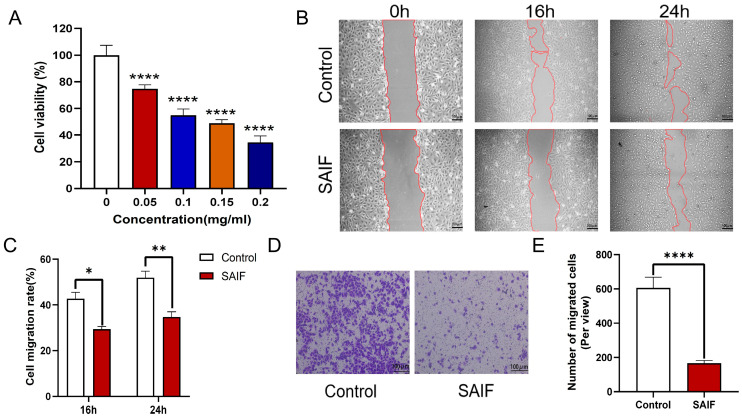
Inhibitory effect of SAIF on HUVEC proliferation and migration. (**A**) Effect of SAIF on the proliferation of HUVEC cells. HUCEV cells were treated with various concentrations of SAIF for 48 h. The CCK8 assay was used to assess the vitality of the cells. (**B**) The migration of HUCEV cells treated with 0.2 mg/mL SAIF for 16 and 24 h was assessed using a scratch assay. (**C**) Image J 1.52a software was used to quantify the cell migration rate in the scratch assay. (**D**) The migration of HUCEV cells treated with 0.2 mg/mL SAIF for 16 and 24 h was assessed using Transwell assay. (**E**) Image J 1.52a software was used to quantify the cell migration rate in the Transwell assay. The results are the mean ± SD values of three experiments performed in triplicate, * *p* < 0.05; ** *p* < 0.01; **** *p* < 0.0001.

HUVEC cells were cultured with SAIF at different doses (0.05, 0.1, 0.15, 0.2 mg/mL) for 48 h. The effect of SAIF on the proliferation of HUVEC cells was detected using the CCK8 assay. Findings indicated that SAIF noticeably suppressed the proliferation of HUVEC cells (*p* < 0.0001). The inhibitory effect on HUVEC cell growth was more pronounced at higher concentrations of SAIF. SAIF showed 65.51% growth inhibition on the HUVEC cells at the highest dose of 0.2 mg/mL (Figure 1A).

Using the scratch and Transwell assays, the impact of SAIF on the migration of HUVEC cells was assessed. The cells were treated with 0.2 mg/mL SAIF for 24 h, with PBS as a control, and then pictures were taken at three time points: 0 h, 16 h, and 24 h (Figure 1B). At 16 h, the scratch-healing rate of the SAIF group was 29.35%, which was notably lower than that of the control group: 42.75% (*p* < 0.05); at 24 h, the scratch-healing rate of the SAIF group was 34.76%, which was also considerably lower compared with the control group: 51.91% (*p* < 0.01) (Figure 1C).

The Transwell assay results showed that compared with the control group, the migration capacities of HUVEC cells in the SAIF group drastically diminished (*p* < 0.0001) (Figure 1D,E). These findings demonstrated that SAIF greatly suppressed the proliferation and migration of HUVEC cells.

### 3.2. SAIF Inhibits the Tube Formation of HUVEC Cells

**Figure 2:**
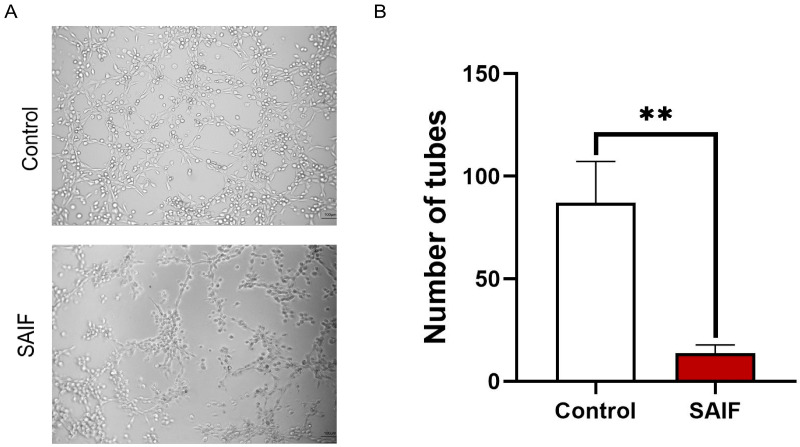
Inhibitory effect of SAIF on HUVEC tube formation. (**A**) HUVEC cells were treated with SAIF for 6 h. The tube formation in HUVEC was observed under microscopy. (**B**) The number of tubes formed by HUVECs was counted by ImageJ 1.52a software. The mean ± SD of three separate experiments is used to express the values, ** *p* < 0.01.

HUVEC cells were treated with 0.2 mg/mL SAIF for 6 h. The number of tubes were counted in randomly selected fields under microscopy. SAIF significantly reduced the number of tubes (*p* < 0.01) compared with the control group, with an inhibition rate of 84.3% (Figure 2A,B). These findings suggested that SAIF considerably suppressed the tube formation of HUVEC cells in vitro.

### 3.3. SAIF Inhibits Vascular Neovascularization of Matrigel Plugs in Mice

**Figure 3:**
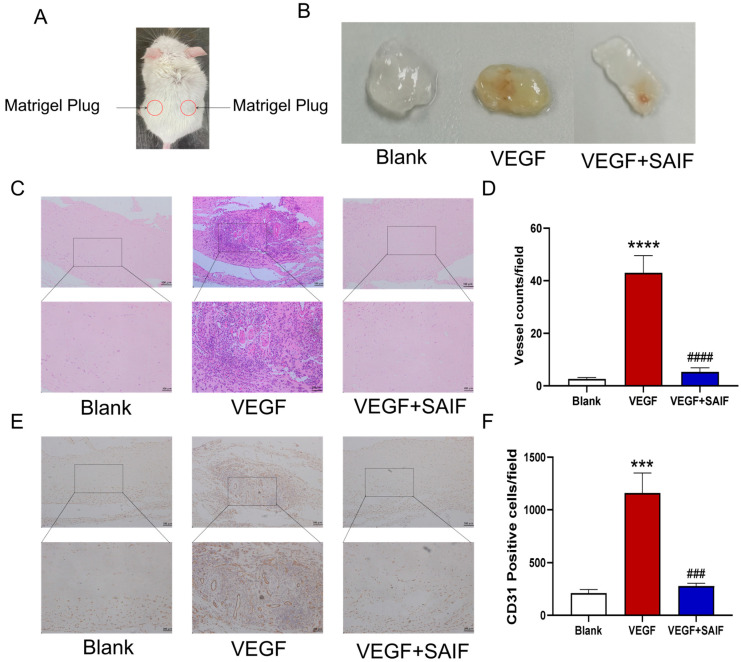
SAIF inhibits exogenous VEGF-induced vascular neovascularization of matrigel plugs in mice. (**A**) Diagram of the matrigel plugs in mice. (**B**) The mixed matrigel plugs containing SAIF (0.2 mg/mL) and VEGF (250 ng/mL) were injected subcutaneously into the dorsal side of the mice and were taken for photographs after 7 days. (**C**) Representative images of H&E staining of matrigel plugs from mice (100×, 200×). (**D**) The number of tubes in the matrigel plugs is presented in the form of a graph. (**E**) Immunohistochemistry staining for CD31 in the matrigel plugs of mice (100×, 200×). (**F**) The graph illustrates the total number of CD31-positive cells. The values are the mean ± SD values of three experiments performed in triplicate. *, for blank vs. VEGF group, *** *p* < 0. 001; **** *p* < 0.0001; #, for VEGF vs. VEGF + SAIF group, ### *p* < 0.001; #### *p* < 0.0001.

The mice were randomly divided into three groups (*n* = 5 mice per group): the blank control group (0.9% NaCl), the positive control group (250 ng/mL VEGF + 50 IU/mL heparin), and the SAIF group (0.2 mg/mL SAIF + 250 ng/mL VEGF + 50 IU/mL heparin). Matrigel plugs were injected subcutaneously into the dorsal side of the mice and then collected after seven days. The experimental procedure is represented in Figure 3A.

The results demonstrated that compared with the blank control group, the matrigel plugs in the VEGF group appeared bright red and were characterized by a large number of neovessels distributed throughout; however, the matrigel plugs in the SAIF group exhibited only a small number of blood vessels under the same experimental condition (Figure 3B). The results of Hematoxylin and Eosin (HE) staining results revealed a significant increase in the number of tubes within the matrigel plugs of the VEGF group compared to the blank control group (*p* < 0.0001); the number of tubes decreased remarkably after SAIF treatment compared to that of the VEGF group (*p* < 0.0001) (Figure 3C,D). The immunohistochemical findings demonstrated that compared with the blank control group, the number of CD31-positive cells (*p* < 0.001) in the matrigel plugs of the VEGF group notably increased and that (*p* < 0.001) in the SAIF group decreased remarkably compared to that of the VEGF group (Figure 3E,F). These findings indicated that SAIF inhibits VEGF-induced neovascularization in vivo.

### 3.4. SAIF Inhibits Corneal Neovascularization in Mice

**Figure 4:**
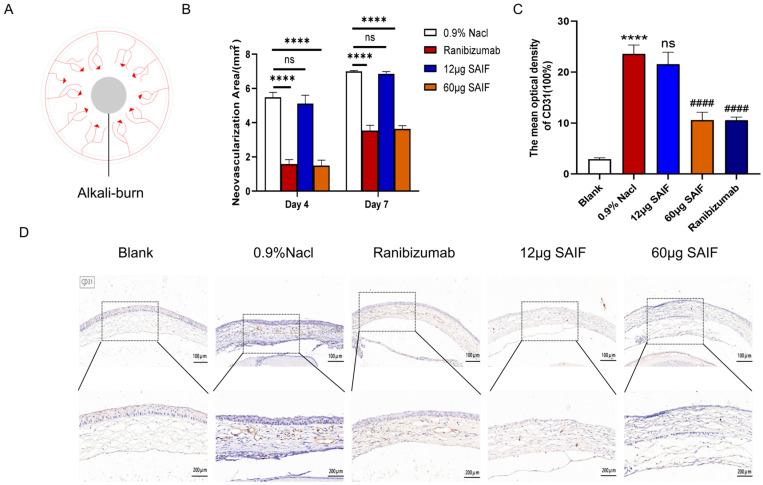
SAIF inhibits corneal neovascularization in mice. (**A**) Chemical injury model induced by alkali burns. The center of the cornea was covered with filter paper sheets that had been soaked in 1 mol/L sodium hydroxide or saline for 30 s and then rinsed with saline. Corneal angiogenesis was observed 4–7 d after alkali burns. (**B**) Corneas of mice after alkali injury were treated with 0.9% NaCl, Ranibizumab, low-dose SAIF (12 μg), and high-dose SAIF (60 μg) and photographed at 4 d and 7 d after administration, and the area of the mouse corneal neovascular network was assessed by ImageJ 1.52a software. **** *p* < 0.0001; ns. No significant difference. (**C**,**D**) Immunohistochemistry staining in the cornea of mice (100×, 200×) and the mean optical densities of CD31 measured by ImageJ 1.52a software. *, for blank vs. 0.9% NaCl group, **** *p* < 0.0001; #, for the drug treatment vs. 0.9% NaCl group, #### *p* < 0.0001; ns. no significant difference. The mean ± SD of three experiments was used to express the values.

The mouse cornea was cauterized to stimulate the regeneration of new blood vessels at the corneal limbus using filter paper sheets soaked in an alkaline solution. The experimental manipulation is shown in Figure 4A. The drug was administered via subconjunctival injection. The blank control group received no treatment. All alkali-burned mice were randomly separated into four groups (*n* = 9): the negative control group (0.9% NaCl, 20 μL); the positive control group (Ranibizumab, 10 μL); the high-dose group: 60 μg of SAIF (3 mg/mL, 20 μL); the low-dose group: 12 μg of SAIF (0.6 mg/mL, 20 μL). Photographs were taken at 4 d and 7 d after administration to count the area of the corneal neovascular network in mice.

The results showed that at 4 d after administration, a large number of new blood vessels sprouted from the corneal limber artery in the 0.9% NaCl group, and the area of the neovascular network was 5.48 mm^2^; the area of the neovascular network in the low-dose 12 μg SAIF group was 5.12 mm^2^. There was no apparent difference between the low-dose SAIF and the 0.9% NaCl group. However, the areas of the neovascular network in the high-dose 60 μg SAIF group and the Ranibizumab group were 1.49 mm^2^ (*p* < 0.0001) and 1.58 mm^2^ (*p* < 0.0001), respectively, and were notably smaller compared with the area of the 0.9% NaCl group.

Continuous vascular development can be observed in the cornea of all groups at 7 d after administration. The areas of the neovascular network in the 0.9% NaCl group and the low-dose 12 μg SAIF group were 6.99 mm^2^ and 6.85 mm^2^, respectively. There was no noticeable difference between these two groups. The areas of the neovascular network in the high-dose 60 μg SAIF group and the positive control group were 3.64 mm^2^ (*p* < 0.0001) and 3.53 mm^2^ (*p* < 0.0001), respectively, and were notably smaller compared with the area of the negative control group (Figure 4B and Figure S1).

Then, we examined the protein expression level of CD31 in the cornea by immunohistochemistry in order to assess the neovascularization. The study results indicated that alkali injury significantly increased the protein expression of CD31 compared to the control group (*p* < 0.0001). The protein expression of CD31 did not differ between the groups treated with 12 μg SAIF and 0.9% NaCl. The CD31 protein expression of the 60 μg SAIF (*p* < 0.0001) and Ranibizumab (*p* < 0.0001) treatment group was lower than that of the 0.9% NaCl group (Figure 4C,D). These results are consistent with those of the tube experiments above.

### 3.5. SAIF Affects the Expression of Angiogenic Factor in Mouse Corneal

The typically inactive vascular system (corneal avascularity) can be activated to sprout new capillaries (corneal angiogenesis), which are controlled by angiogenic switch mechanisms [21]. Disruption of the balance between pro-angiogenic and anti-angiogenic factors due to various injuries can lead to corneal neovascularization [8]. To further explore the role of SAIF in inhibiting corneal neovascularization, the transcript levels of the pro-angiogenic factors VEGF (vascular endothelial growth factor), bFGF (basic fibroblast growth factor), PDGF (platelet-derived growth factor), and the anti-angiogenic factor PEDF (pigment epithelium-derived factor) were detected in mouse corneal tissues by qRT-PCR.

**Figure 5:**
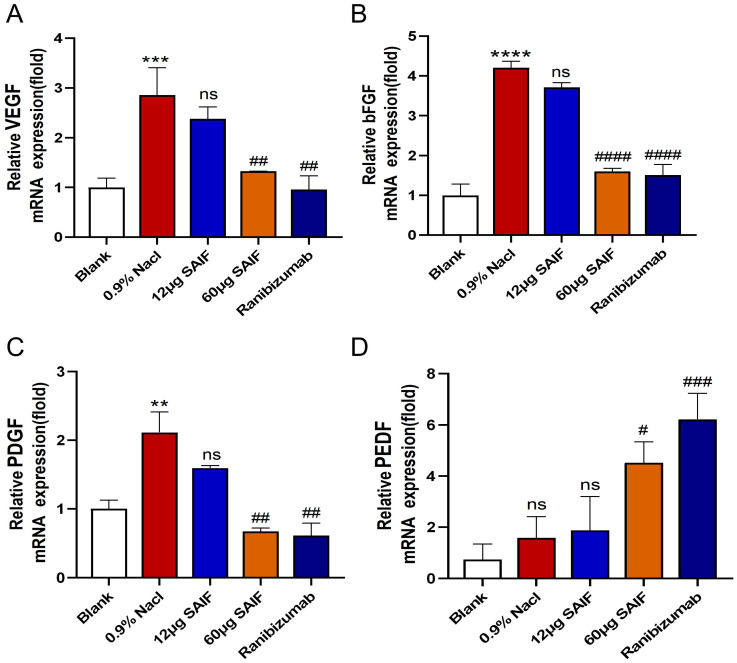
SAIF affects the mRNA expression of angiogenic factors in mouse corneal. (**A**–**D**) Mouse corneas were taken and RNA was extracted for qRT-PCR to assess the mRNA expression levels of angiogenic factors VEGF, bFGF, PDGF, and PEDF. Values are the mean ± SD values of three experiments performed in triplicate, for blank vs. 0.9% NaCl group, ** *p* < 0.01; *** *p*< 0.001; **** *p* < 0.0001; ns. no significant difference; #, for the drug treatment vs. 0.9% NaCl group, #, *p* < 0.05; ## *p* < 0.01; ### *p* < 0.001. #### *p* < 0.0001; ns. no significant difference.

Findings demonstrated that the mRNA expression level of the pro-angiogenic factors VEGF (*p* < 0.001), bFGF (*p* < 0.0001), and PDGF (*p* < 0.01) in the 0.9% NaCl group were upregulated compared with the blank group. The transcript levels of the pro-angiogenesis-related genes did not differ between the 0.9% NaCl group and the 12 μg SAIF treatment group. Compared with the 0.9% NaCl group, the mRNA expression of VEGF (*p* < 0.01), bFGF (*p* < 0.0001), and PDGF (*p* < 0.01) was notably lower in the 60 μg SAIF treatment group. The mRNA expression of the VEGF (*p* < 0.01), bFGF (*p* < 0.0001), and PDGF (*p* < 0.01) in the Ranibizumab group was also considerably lower compared with the 0.9% NaCl group. (Figure 5A–C).

Compared with the blank group, the mRNA expression level of PEDF in the 0.9% NaCl group showed an upregulated trend, but there was no noticeable distinction. There also was no difference between the 0.9% NaCl group and the 12 μg SAIF treatment group in the mRNA expression of PEDF. Compared with the 0.9% NaCl group, the transcript level of PEDF (*p* < 0.05) in the 60 μg SAIF treatment group was significantly upregulated. The mRNA expression of PEDF (*p* < 0.001) was also significantly higher after Ranibizumab treatment compared with the 0.9% NaCl group (Figure 5D).

**Figure 6:**
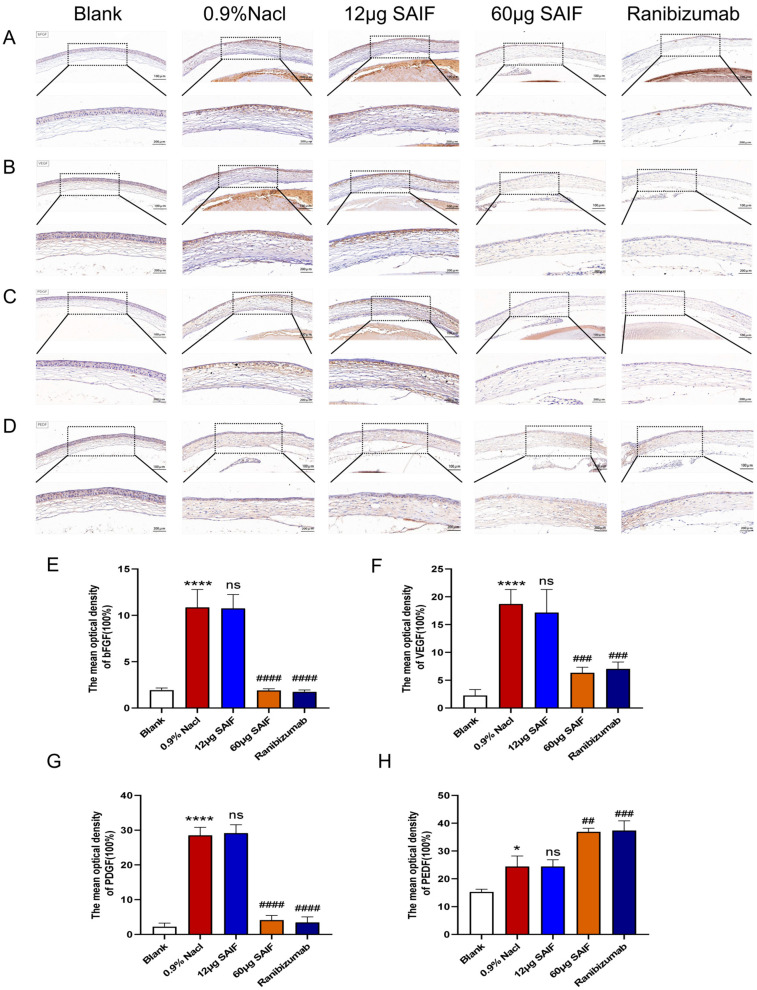
SAIF affects the protein expression of angiogenic factors in mouse corneas. Immunohistochemistry staining in the cornea of mice (100×, 200×) and the mean optical densities of VEGF (**A**,**E**), bFGF (**B**,**F**), PDGF (**C**,**G**), and PEDF (**D**,**H**) measured by ImageJ 1.52a software. Results are the mean ± SD values of three experiments performed in triplicate. *, for the blank vs. 0.9% NaCl group, * *p* < 0.05; **** *p* < 0.0001; #, for drug treatment vs. 0.9% NaCl group, ## *p* < 0.01; ### *p* < 0.001; #### *p* < 0.0001; ns. No significant difference.

Then, we investigated the protein expression of angiogenic factors in the cornea by immunohistochemistry after SAIF treatment. The findings demonstrated that alkali injury markedly upregulated the protein expression level of pro-angiogenic factors VEGF (*p* < 0.0001), bFGF (*p* < 0.0001), and PDGF (*p* < 0.0001) in the cornea compared with the blank group. The protein expression of the pro-angiogenic factors did not differ between the 0.9% NaCl group and the 12 μg SAIF treatment group. The protein expression of VEGF (*p* < 0.0001), bFGF (*p* < 0.001), and PDGF (*p* < 0.0001) in the 60 μg SAIF treatment group was considerably lower compared with the 0.9% NaCl group. Ranibizumab administration significantly reduced the protein expression of VEGF (*p* < 0.0001), bFGF (*p* < 0.001), and PDGF (*p* < 0.0001) compared to the 0.9% NaCl group (Figure 6A–C,E–G).

The protein expression level of PEDF in the 0.9% NaCl group was notably upregulated compared to that of the blank group (*p* < 0.01). The protein expression of PEDF did not differ significantly between the 12 μg SAIF treatment group and the 0.9% NaCl group. The protein expression of PEDF in the 60 μg SAIF treatment group (*p* < 0.01) was significantly higher compared with the 0.9% NaCl group. The protein expression of PEDF (*p* < 0.001) in the Ranibizumab treatment group was also significantly higher than that of the 0.9% NaCl group (Figure 6D,H).

### 3.6. SAIF Affects the Expression of MMPs in Mouse Corneal

**Figure 7:**
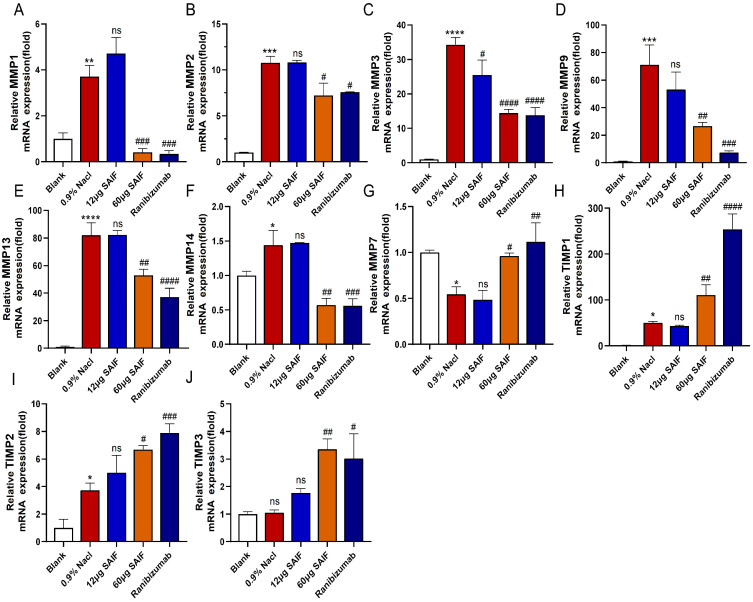
SAIF affects the expression of MMPs in mouse corneal. (**A**–**J**) Mouse corneas were collected and RNA was extracted for qRT-PCR to detect the mRNA expression levels of MMP1, MMP2, MMP3, MMP7, MMP9, MMP13, MMP14, TIMP1, TIMP2, and TIMP3. The mean ± SD of three separate experiments were used to express the values. *—the blank vs. the 0.9% NaCl group, * *p* < 0.05; ** *p* < 0.01; *** *p* < 0.001; **** *p* < 0.0001; ns. no significant difference; #, the drug group vs. the 0.9% NaCl group, # *p* < 0.05; ## *p* < 0.01; #### *p* < 0.001; #### *p* < 0.0001; ns. no significant difference.

MMPs are a group of zinc-binding protein hydrolases involved in extracellular matrix (ECM) remodeling, angiogenesis, and lymphangiogenesis [22]. The transcript levels of MMPs in mouse corneas were assessed by qRT-PCR. Findings demonstrated that compared with the blank group, the mRNA expression levels of MMP1 (*p* < 0.01), MMP2 (*p* < 0.001), MMP3 (*p* < 0.0001), MMP9 (*p* < 0.001), MMP13 (*p* < 0.0001), and MMP14 (*p* < 0.05) after alkaline damage were significantly upregulated. Except for MMP3, the mRNA of other MMPs did not differ substantially between the 12 μg SAIF treatment group and the 0.9% NaCl group. Compared with the 0.9% NaCl group, the mRNA expression of MMP1 (*p* < 0.001), MMP2 (*p* < 0.05), MMP3 (*p* < 0.0001), MMP9 (*p* < 0.01), MMP13 (*p* < 0.01), and MMP14 (*p* < 0.01) in the 60 μg SAIF treatment group were significantly lower. The transcript levels of MMP1 (*p* < 0.001), MMP2 (*p* < 0.05), MMP3 (*p* < 0.0001), MMP9 (*p* < 0.001), MMP13 (*p* < 0.0001), and MMP14 (*p* < 0.001) in the Ranibizumab treatment group were also notably downregulated than that of the 0.9% NaCl group (Figure 7A–F).

MMP-7 cleaves corneal collagen XVIII to produce an endostatin-spanning fragment capable of inhibiting angiogenesis, which is crucial in preserving corneal avascularity [23]. Alkali injury significantly downregulated the transcript level of MMP7 in the cornea compared with the blank group (*p* < 0.05). The mRNA expression of MMP7 did not significantly differ between the 12 μg SAIF treatment group and the 0.9% NaCl group. Both the transcript level of MMP7 in the 60 μg SAIF treatment (*p* < 0.05) and Ranibizumab treatment group (*p* < 0.01) were considerably upregulated compared with the 0.9% NaCl group (Figure 7G). These findings suggested that SAIF suppresses corneal angiogenesis in mice by modulating the expression of MMPs.

TIMPs, a group of metalloproteinase tissue inhibitors, play a role in extracellular matrix remodeling by controlling the activity of MMPs and are likewise important determinants of corneal neovascularization [24]. qRT-PCR results demonstrated that alkali injury notably upregulated the transcript levels of TIMP1 (*p* < 0.05) and TIMP2 (*p* < 0.05) in mouse corneas compared with the blank group. However, the TIMP3 mRNA expression did not differ notably between the 0.9% NaCl and the blank group. The mRNA levels of TIMP1, TIMP2, and TIMP3 showed no significant variation between the 0.9% NaCl group and the 12 μg SAIF treatment group. The mRNA expression of TIMP1 (*p* < 0.01), TIMP2 (*p* < 0.01), and TIMP2 (*p* < 0.01) in the 60 μg SAIF treatment group was considerably higher compared with the 0.9% NaCl group. The transcript levels of TIMP1 (*p* < 0.01), TIMP2 (*p* < 0.05), and TIMP3 (*p* < 0.01) in the Ranibizumab treatment group were also considerably upregulated compared to those of the 0.9% NaCl group (Figure 7H–J). These results indicated that SAIF inhibits corneal angiogenesis in mice by promoting the expression of TIMPs.

## 4. Discussion

The cornea, a transparent and avascular tissue with refractive properties, is susceptible to neovascularization-induced loss of transparency, which ranks as the second leading cause of visual impairment and blindness [25]. Clinically available treatments for corneal neovascularization have certain therapeutic limitations and produce side effects, such as risk of infection and neurotoxic effects [8]. The limited market for treating corneal neovascularization highlights the need to develop new drugs. Cartilage, which is typically avascular with a poor blood supply, is a potential source of anti-angiogenic substances [13]. It is reported that shark cartilage extracts possess potent anti-angiogenesis properties that inhibit tumors [17], although their specific mechanisms in addressing corneal neovascularization remain unclear. Our study showed that SAIF significantly inhibited the proliferation, migration, and tube formation of endothelial cells in vitro. The Matrigel plug assay also showed that SAIF attenuated VEGF-induced neovascularization, and SAIF notably reduced the number of tubes and CD31-positive cells compared to the VEGF treatment group. The mouse corneal alkali injury study also identified that 60 μg of SAIF significantly inhibited corneal neovascularization in vivo.

We also investigated the mechanism of SAIF inhibiting corneal neovascularization by analyzing pro-angiogenesis and anti-angiogenesis factors. Several angiogenic factors, including bFGF, VEGF, and PDGF, are involved in corneal neovascularization [8]. VEGF, a well-recognized angiogenic factor, is a key cytokine in tumor neovascularization and retinal neovascularization, and anti-VEGF treatment significantly inhibited corneal neovascularization [26]. The findings suggested that the transcription and protein expression levels of VEGF were upregulated after alkali burns; however, the upregulation of VEGF was largely attenuated by SAIF. bFGF is widely expressed during cellular differentiation, mitosis, and angiogenesis [27]. After corneal injury, bFGF is highly expressed in corneal epithelial, stromal, and inflammatory cells [28]. We also found that SAIF reduced the increase in bFGF mRNA and protein expression in the cornea induced by alkali injury. In addition to VEGF and bFGF, PDGF also plays a crucial role in corneal neovascularization [29]. PDGF is a typical mitogenic growth factor that promotes the proliferation of stem cells to form new blood vessels [30,31]. In this study, SAIF inhibited the upregulation of PDGF mRNA and protein expression in corneal issues induced by alkali injury. The downregulation of VEGF, bFGF, and PDGF mRNA and protein expression in the cornea suggested that SAIF exerts anti-angiogenic activity by inhibiting the expression of pro-angiogenic factors. The preservation of corneal avascularity is not solely attributed to the decrease in pro-angiogenic factors, but also to the increase in anti-angiogenic factors [2,8]. PEDF is a powerful anti-angiogenic factor in the serine protein superfamily [32]. PEDF is highly expressed in several ocular tissues including corneal epithelium and endothelium to maintain the corneal avascular properties [33]. Our study discovered that the mRNA and protein expression levels of PEDF were upregulated in mouse corneas after administration of SAIF, indicating that SAIF inhibits corneal neovascularization by promoting the expression of anti-angiogenic factors.

MMPs, a group of zinc-binding protein hydrolases, are crucial in extracellular matrix remodeling, angiogenesis, and lymphangiogenesis [8]. MMPs exhibit dual functions in angiogenesis; on the one hand, they can degrade the ECM, which allows MMP-carrying endothelial cells to invade tissues and promote neovascularization, and on the other hand, they can produce or release anti-angiogenic fragments from the precursor, exerting anti-angiogenic properties [34]. The delicate balance between corneal angiogenesis and avascularity is partially attributed to 14 MMPs, now categorized as pro-angiogenic and anti-angiogenic molecules [35]. MMP1 and MMP9 are highly expressed in platelet-activating factor-induced corneal neovascularization [36]. The expression of MMP2 and MMP14 is increased in alkaline burn-induced corneal angiogenesis [37]. The MMP3 gene expression exhibited an elevation in the stroma of diabetic retinopathy corneas [38]. Selective MMP13 inhibitors attenuated corneal neovascularization induced by alkali injury [39]. Our study discovered that the mRNA expression of MMP1, MMP2, MMP3, MMP9, MMP13, and MMP14 in the cornea increased following alkali injury, and this increase was significantly reduced by SAIF treatment. MMP-7 can cleave corneal collagen XVIII to produce endostatin-spanning fragment, which exerts anti-angiogenic activity [40]. Compared with age-matched WT mice, corneal neovascularization was significantly increased in MMP-7 KO mice after keratotomy [41]. Our study also observed a notable reduction in the mRNA expression of MMP7 after alkali injury, but with SAIF administration, the MMP7 mRNA expression significantly increased. TIMP is a group of tissue inhibitors of metalloproteinases that prevent the unregulated degradation of corneal stroma and inhibit corneal vascular neogenesis [42]. It is reported that the expression levels of TIMP-1 and TIMP-2 were upregulated in inflammation-induced corneal neovascularization [43], while mice lacking TIMP3 exhibited increased corneal neovascularization [44]. Our study found that SAIF promoted the expression of TIMPs in alkali injury-induced corneal neovascularization. These results indicated that SAIF inhibits corneal neovascularization by regulating the expression of MMPs.

In summary, SAIF acts as an anti-angiogenic factor to inhibit the proliferation, migration, and tube formation of endothelial cells, inhibit pro-angiogenic factors, promote anti-angiogenic factors, and regulate the expression of MMPs, ultimately inhibiting corneal neovascularization.

## Data Availability

The original contributions presented in the study are included in the article/Supplementary Materials, further inquiries can be directed to the corresponding author/s.

## Supplementary Materials

The following supporting information can be downloaded at: https://www.mdpi.com/article/10.3390/bioengineering11070693/s1, Figure S1: SAIF inhibits corneal neovascularisation in mice. Corneas of mice after alkali injury were treated with 0.9% NaCl, Ranibizumab, low-dose SAIF (12 μg), and high-dose SAIF (60 μg) and photographed at 4 d and 7 d after administration.
