# Supplementary material for: Shark Cartilage-Derived Anti-Angiogenic Peptide Inhibits Corneal Neovascularization
Source: Bioengineering (Basel). 2024 Jul 9;11(7):693. doi: 10.3390/bioengineering11070693 (PMC11273382; doi:10.3390/bioengineering11070693)
Supplement: Supplementary file 1 [file bioengineering-11-00693-s001.zip › bioengineering-3064393-supplementary.pdf]

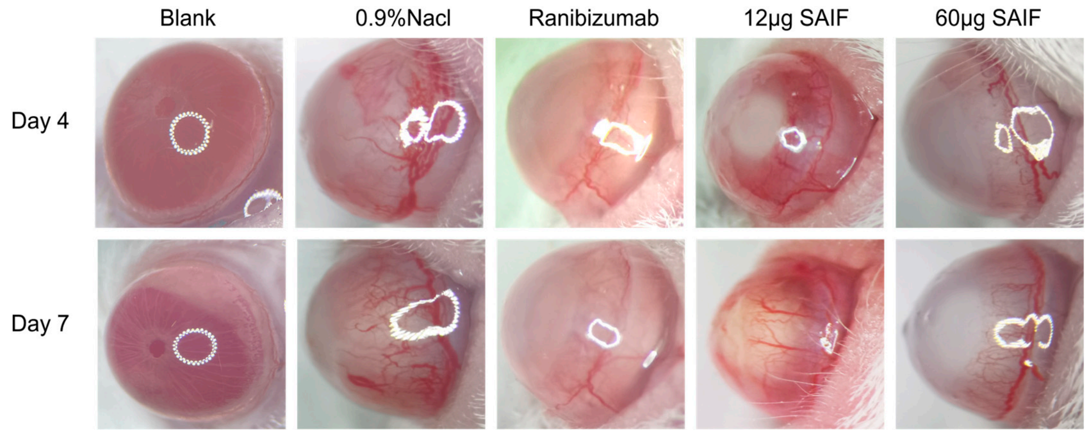

Figure S1. SAIF inhibits corneal neovascularisation in mice. Corneas of mice after alkali injury were treated with 0.9% NaCl, Ranibizumab, low-dose SAIF (12 μg), and high-dose SAIF (60 μg) and photographed at 4 d and 7 d after administration.
